# Ovarian Cancer Translational Activity of the Multicenter Italian Trial in Ovarian Cancer (MITO) Group: Lessons Learned in 10 Years of Experience

**DOI:** 10.3390/cells9040903

**Published:** 2020-04-07

**Authors:** Daniela Califano, Daniela Russo, Giosuè Scognamiglio, Nunzia Simona Losito, Anna Spina, Anna Maria Bello, Anna Capiluongo, Francesca Galdiero, Rossella De Cecio, Simona Bevilacqua, Piera Gargiulo, Edoardo Marchesi, Silvana Canevari, Francesco Perrone, Gennaro Daniele, Loris De Cecco, Delia Mezzanzanica, Sandro Pignata

**Affiliations:** 1 Functional Genomic Unit, Istituto Nazionale Tumori – IRCCS - “Fondazione G. Pascale”, 80131 Naples, Italy; d.califano@istitutotumori.na.it (D.C.); d.russo@istitutotumori.na.it (D.R.); a.spina@istitutotumori.na.it (A.S.); a.bello@istitutotumori.na.it (A.M.B.); a.capiluongo@istitutotumori.na.it (A.C.); f.galdiero@istitutotumori.na.it (F.G.); 2 Surgical Pathology Unit, Istituto Nazionale Tumori – IRCCS - “Fondazione G. Pascale”, 80131 Naples, Italy; giosue.scognamiglio@istitutotumori.na.it (G.S.); n.losito@istitutotumori.na.it (N.S.L.); r.dececio@istitutotumori.na.it (R.D.C.); 3 Clinical Trials Unit, Istituto Nazionale Tumori – IRCCS - “Fondazione G. Pascale”, 80131 Naples, Italy; s.bevilacqua@istitutotumori.na.it (S.B.); piera.gargiulo@istitutotumori.na.it (P.G.); f.perrone@istitutotumori.na.it (F.P.); gennaro.daniele@policlinicogemelli.it (G.D.); 4 Integrated Biology Platform, Fondazione IRCCS Istituto Nazionale dei Tumori Milan, 20133 Milan, Italy; edoardo.marchesi@istitutotumori.mi.it (E.M.); loris.dececco@istitutotumori.mi.it (L.D.C.); 5 Molecular Therapies Unit, Fondazione IRCCS Istituto Nazionale dei Tumori Milan, 20133 Milan, Italy; silvana.canevari@istitutotumori.mi.it (S.C.); delia.mezzanzanica@istitutotumori.mi.it (D.M.); 6 Urogynaecological Medical Oncology Unit Istituto Nazionale Tumori – IRCCS - “Fondazione G. Pascale”, 80131 Naples, Italy

**Keywords:** translational studies design, gynecological cancers, biomarkers definition, precision medicine, FFPE block collection

## Abstract

Ovarian cancer is the most lethal gynecological cancer, and despite years of research, with the exception of a BRCA mutation driving the use of PARP inhibitors, no new prognostic/predictive biomarkers are clinically available. Improvement in biomarker selection and validation may derive from the systematic inclusion of translational analyses into the design of clinical trials. In the era of personalized medicine, the prospective centralized collection of high-quality biological material, expert pathological revision, and association to well-controlled clinical data are important or even essential added values to clinical trials. Here, we present the academic experience of the MITO (Multicenter Italian Trial in Ovarian Cancer) group, including gynecologists, pathologists, oncologists, biostatisticians, and translational researchers, whose effort is dedicated to the care and basic/translational research of gynecologic cancer. In our ten years of experience, we have been able to collect and process, for translational analyses, formalin-fixed, paraffin-embedded blocks from more than one thousand ovarian cancer patients. Standard operating procedures for collection, shipping, and processing were developed and made available to MITO researchers through the coordinating center’s web-based platform. Clinical data were collected through dedicated electronic case report forms hosted in a web-based electronic platform and stored in a central database at the trial’s coordinating center, which performed all the analyses related to the proposed translational researches. During this time, we improved our strategies of block management from retrospective to prospective collection, up to the design of a prospective collection with a quality check for sample eligibility before patients’ accrual. The final aim of our work is to share our experience by suggesting a guideline for the process of centralized collection, revision processing, and storing of formalin-fixed, paraffin-embedded blocks for translational purposes.

## 1. Introduction

The molecular characterization of epithelial ovarian cancer (EOC) in order to select or drive new drug development is perceived as a major goal by all the stakeholders in the field [1]. Despite years of research and the publication of hundreds of potential bio-markers, with the exception of BRCA, no new prognostic or predictive biomarkers have been validated in EOC [2]. Among the potential flaws hindering the development of translational research in this field there are samples sizes, missing or imprecise clinical information, and a lack of rigorous statistical analysis. In our view, some of these limitations might be overcome by designing translational analyses together with clinical trials [3]. The collection of samples from clinical trials is a precious source for translational studies; examples of such bio-banking include the International Breast Cancer Study Group (IBCSG) Biobank, which contains formalin-fixed paraffin-embedded primary breast cancer blocks from over 30,000 patients included in clinical trials conducted by the Group since 1981 (http://www.ibcsg.org), and the NHI-NCI experience, with a collection of samples from over 61,000 patients enrolled in NCI’s trials (http://navigator.ctsu.org). However, very few of these initiatives are conducted by academic institutions or group in a multicenter setting, mainly due to the scarcity of finances and complexity of the procedures. The accomplishment of this goal is one of the aims of the MITO (Multicenter Italian Trial in Ovarian Cancer), a national cooperative group encompassing more than 480 clinicians (gynecologists, pathologists, oncologists) from more than 60 Italian clinical centers focused on the care of gynecologic cancer patients (www.mito-group.it/en/). MITO acts within the European Network for Gynecological Oncological Trial (https://engot.esg.org) and the Gynecological Cancer Inter Group (www.gcigtrials.gov) conducting also international trials. Since 2010, a translational group has been established within MITO, comprised of 15 laboratories involved in basic and translational research in gynecologic cancer. This led, starting from the MITO2 [4], to include a translational research plan in all the academic trials promoted by the MITO group. During these years, the management of formalin-fixed, paraffin-embedded (FFPE) blocks’ collection and processing evolved in line with the complexity of the clinical trials, from retrospective to prospective and prospective with quality check for sample eligibility. With the aim of ensuring a high quality of FFPE blocks and useful molecular and statistical analyses, we developed and refined a number of procedures related to the process of centralized collection, revision processing, and the storing of formalin-fixed paraffin embedded blocks that we describe here, in order to facilitate other groups interested in performing translational research in this field.

## 2. Materials and Methods

### 2.1. Trials and Patients 

We reported the translational activities associated with four MITO (Multicenter Italian Trial in Ovarian Cancer) trials, coordinated by the Clinical Trials Unit at Istituto Nazionale Tumori IRCCS "Fondazione G. Pascale" of Naples, and designed to evaluate treatment optimization for advanced EOC patients (Table 1).

FFPE blocks were collected from patients enrolled in MITO2 [4], MITO7 [5], MITO16A/MaNGO-OV2 (EudraCT number: 2012-003043-29; hereafter indicated as MITO16A), and MITO16B/MaNGO-OV2B/ENGOT-OV17 (EudraCT number: 2012-004362-17; hereafter indicated as MITO16B) clinical trials (for eligibility criteria, see references to each study). In each translational study, approved by ethical committees, the goals of the study, the proposed molecular analyses, the type of biological sample to be collected, and the size of samples required for analysis were defined.

All patients signed an informed consent form before entering into each trial. For retrospective translational studies, amendments were presented and approved by ethical committee, while for prospective translational studies a specific informed consent for translational research was provided to patients at the enrollment in the study. With respect to regulatory rules, patients’ privacy was guaranteed by assigning an anonymized ID to each patient at entrance into the study. Clinical data were collected through dedicated electronic case report forms (eCRFs) hosted in the web-based electronic platform (www.usc-intnapoli.net) and stored in a central database at the coordinating center.

### 2.2. FFPE Block Collection 

Formalin-fixed, paraffin-embedded (FFPE) blocks were collected and stored according to Italian guidelines [6] and dedicated standard operating procedures (SOPs) for collection, shipping, and processing were developed and made available to MITO researchers through the coordinating center’s web-based platform (Supplementary Figure S1). The coordinating center collected FFPE blocks from each local center, with an anonymized copy of the pathology report, a hematoxylin and eosin (H&E) stained slide, when available, and a filled shipping log. For trials with a prospective collection of biological FFPE blocks, shipment was scheduled by the coordinating center either for every 10 patients enrolled or every 2 months. The identity of the blocks received and concordance with the eCRF was verified by the coordinating center, and in the case of inconsistency, a specific query was issued to the peripheral center. The FFPE blocks were identified with the MITO patient’s ID and stored under ambient conditions until processing. 

In cases where peripheral centers asked for a sample to be sent back in the patient’s interest (trial participation, pathology second opinion etc.), whenever possible sections were provided in order to keep the block for the proposed translational analysis.

### 2.3. Pathological Revision 

Pathological revision of centralized FFPE blocks was usually performed before processing, except for some of the blocks of MITO2 case material (see below). If not provided by the peripheral centers, a 5 μm section was cut from each FFPE block, stained with H&E, and reviewed by a team of gynecology expert pathologists; in the case of discordant diagnosis, appropriate immunohistochemical (IHC) stains were performed to exclude wrong diagnoses, such as secondary metastatic localizations or borderline cases; moreover, a query was issued to the peripheral pathologist. Only blocks with a concordant diagnosis were further processed. The result of the revision came with the following information: histology type, grading, cancer site, selection of representative tumor area in the section, percentage of tumor cells, and percentage of necrosis. Only chemo-naïve blocks were considered adequate for the proposed analyses, and the primary localization of the tumor was preferential.

### 2.4. FFPE Blocks Processing

**Figure 1:**
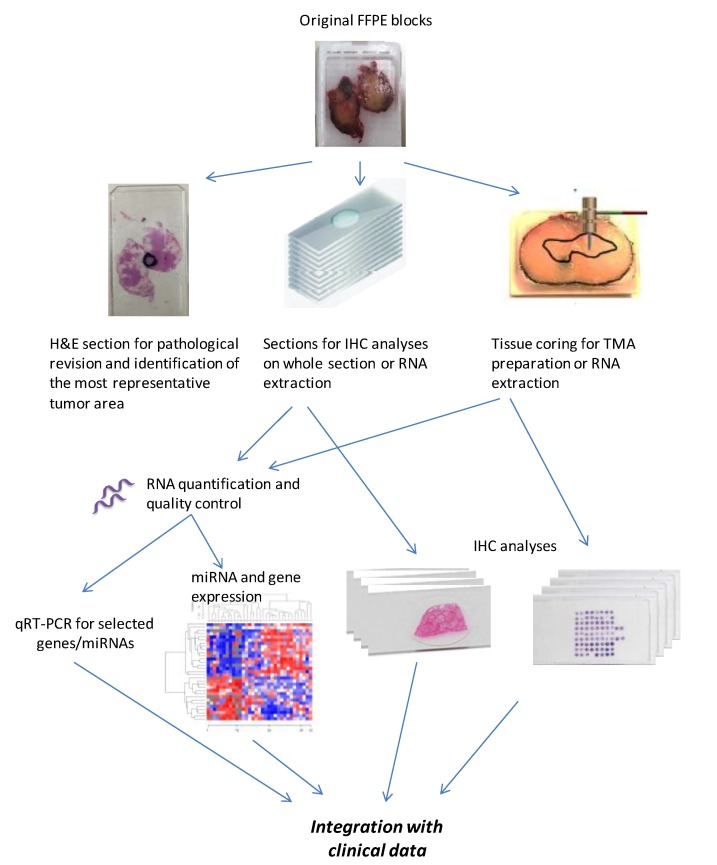
Schematic representation of formalin-fixed, paraffin-embedded (FFPE) block processing for translational analyses associated with the Multicenter Italian Trial in Ovarian Cancer (MITO) clinical trials.

The schematic representation of FFPE block processing is reported in Figure 1. FFPE collected blocks were processed for IHC analyses or nucleic acid extraction, giving priority to IHC analyses in the case of a limited amount of adequate material. In the case of a motivated and approved request for IHC evaluation of specified biomarkers on a whole section, 4 µm sections from 25 original FFPE blocks were cut weekly and sent to recipient labs. In all the other cases, IHC analyses were performed on tissue microarrays (TMAs) prepared from FFPE blocks.

### 2.5. TMA Building for IHC Analysis 

TMAs were built taking the most representative areas from each single case. Two (MITO2 and MITO7) or three (MITO16A and B) 1 mm cores were collected from each eligible tumor block and arrayed into a recipient paraffin block (35 mm × 20 mm) using a semiautomatic tissue array instrument (Galileo CK3500 TMA, ISENET, Milan, Italy). The criteria applied in TMA building are summarized in Supplementary Table S1.

For IHC analysis of each biomarker, 4 μm-thick, non-consecutive sections were cut from TMAs to respect vertical heterogeneity, and were sent to the centers according to the approved project.

### 2.6. RNA Extraction and Quality Controls 

From MITO2 and MITO7 FFPE blocks, the total RNA was extracted from 20 μm-thick whole sections or dissected macro-areas, according to the tumor size, and only from blocks with more than 70% tumor cells and less than 20% necrosis. For MITO16 trials, due to the largest number of planned molecular analyses, the extraction was performed from two 1 mm cores of FFPE tissues, with no quantitative or qualitative differences between RNA extracted from whole sections or from representative cores (Figure 1). RNA was extracted using Qiagen miRNeasy FFPE Kit with a partially modified protocol, as specified in the Supplementary Materials. After extraction, the median amount of extracted RNA was 1–6 μg RNA/mm^3^ tissue. RNA concentration was assessed by the Nanodrop 2000 UV-Vis spectrophotometer, and samples with RNA concentration lower than 80 ng/µL were excluded due to the lack of material for a second extraction. RNA stock samples were stored at −80 °C, undiluted, and an aliquot (2.5 µg) was delivered to the Fondazione IRCCS Istituto Tumori Milan for quality control. Sample purity was assessed by 260/280 and 260/230 ratios, and the recorded values ranged from 1.9–2.2.

The RNA integrity was evaluated by real-time PCR amplification of four miRNAs considered constitutively over-expressed in tumors and four housekeeping genes (see details in the Supplementary Materials). Only RNA of adequate quality was then sent to the centers, according to the approved projects. The effects of batch of RNA extraction and the FFPE blocks’ age on the quality of extracted RNA was assessed by a Kruskal–Wallis test and by unsupervised hierarchical clustering analysis, using the ComplexHeatmap R package available in the Bioconductor repository, with Pearson’s correlation as distance metrics [7].

## 3. Results

### 3.1. FFPE Block Collection

In Supplementary Figures S2–S5 the flow diagrams of sample collection/dropout for the translational analyses associated with each one of the four clinical trials are depicted.

For MITO2 (Supplementary Figure S2), 17/43 centers (39%) accepted participating in the biological study, with 549 patients (67% of the whole study); however, adequate FFPE blocks were supplied from only 269 patients. After pathological revision, 30 patients were excluded because their tumors were collected during interval debulking surgery after neo-adjuvant chemotherapy (NACT), or because the date of collection was unknown, and a further 25 were excluded because tumor blocks did not fulfill the criteria for RNA extraction. Overall, FFPE blocks from 239 patients were eligible for IHC analyses and 214 for RNA extraction. 

For MITO7 (Supplementary Figure S3), 12/67centers (18%) accepted participating in the biological study, with 457/810 patients (56% of the whole study); however, adequate FFPE blocks were supplied from 176 patients only (22%). Drop-outs subsequent to pathological revision were due to withdrawn consent (1 patient); exclusion because tumors were collected after NACT, or because the date of collection was unknown (12 patients); and an insufficient amount of tumor tissue for IHC/RNA extraction or fixation in Bouin rather than formalin (27 patients). Blocks from 158 patients (19%) were therefore available for IHC analyses, and blocks from 136 patients (17%) were eligible for RNA extraction.

For MITO16A (Supplementary Figure S4), 47 centers enrolling 400 patients supplied FFPE blocks from the basal surgery or diagnostic biopsies of 385 (96%) patients. Following pathological revision, the following drop-outs were recorded: exclusion because tumors were collected after NACT (12 patients); insufficient amount of tumor tissue for IHC/RNA extraction (15 patients); and blocks were inadequate or had insufficient material for RNA extraction (68 patients). Therefore, for 358 patients (90%), the tumor blocks were adequate for IHC analyses, and blocks from 290 patients (72%) were eligible for RNA extraction. 

For MITO16B (Supplementary Figure S5), 82 centers enrolling 406 patients supplied FFPE blocks of 366 patients (90%)—for 40 patients, tumor tissues were not available. Following pathological revision, the following drop-outs were recorded: exclusion because tumors were collected after NACT (33 patients); insufficient amount of tumor tissue for IHC/RNA extraction (20 patients); and inadequate or insufficient tumor tissue for RNA extraction (57 patients). Therefore, for 313 patients (77%), blocks were available for IHC analyses, and blocks from 256 patients (63%) were eligible for RNA extraction.

### 3.2. Performance of FFPE Block Collection over 10 Years of Activity 

In Table 2 is reported the performance of block collection over time.

**Figure 2:**
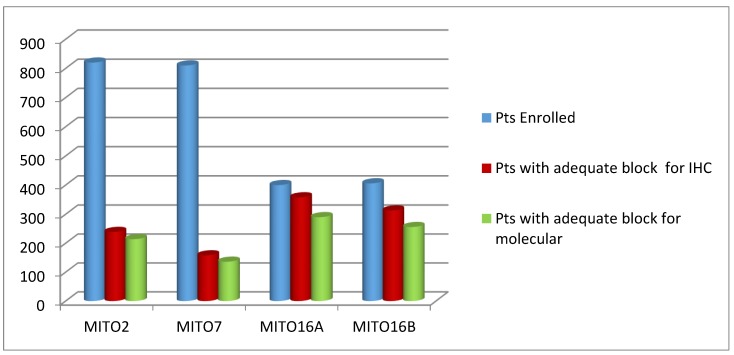
Number of patients (Pts) enrolled in the four clinical trials, with FFPE blocks available/adequate for the indicated purposes. Block collection was retrospective for MITO2 and MITO7, and prospective for MITO16A/B.

Moving from a retrospective to a prospective collection of FFPE blocks, the percentage of patients included into translational analyses was greatly increased (Figure 2).

The apparent decrease in percentage of blocks adequate for molecular analysis in the two MITO16 trials is due to the decision to use representative tumors’ cores rather than whole section for RNA extraction, and cores with more than 70% tumor and less than 20% necrosis were not always present. This decision was due to the increased number of proposed molecular analyses in MITO16 translational projects. This procedure did not negatively impact on the quality of the studies. It has to be noted that for some of the patients entering the trials, synchronous FFPE blocks from the primary tumor and from peritoneal secondary localizations were received, while for some others only secondary localizations were available (Table 3), although the primary tumor was the preferential site requested for translational analyses. 

**Figure 3:**
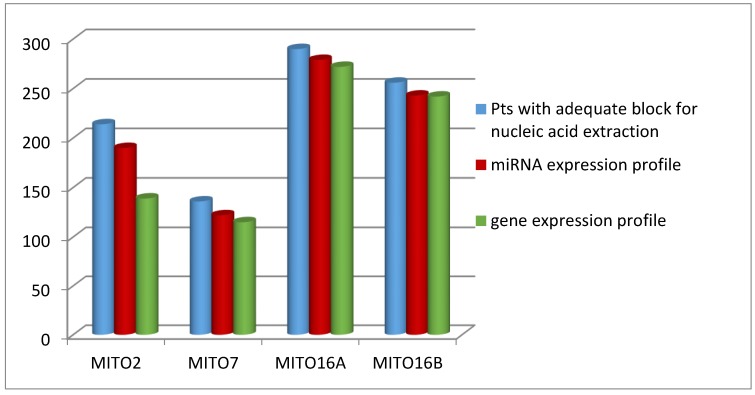
Number of patients (Pts) with adequate blocks for RNA extraction and with the quality of RNA adequate for the indicated analyses.

Particular attention was given to the quality of extracted RNA, since the low quality of this nucleic acid could greatly affect the results of molecular analysis, in particular gene expression profiles for which the integrity of RNA is of particular relevance. We were able to increase the quality of extracted RNA over time. Indeed, while for MITO2 case material the RNA was of adequate quality in the 65% of the extracted cases, for MITO16 case materials we obtained RNA of excellent quality in over 95% of the extracted cases (Figure 3). 

**Figure 4:**
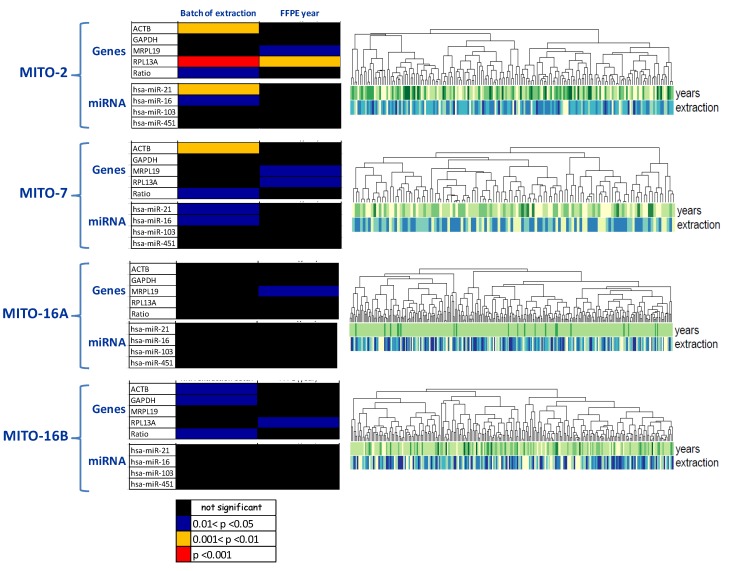
Pre-analytical systematic effects influencing the quality of RNA extracted from FFPE blocks of the four selected MITO trials. **Left**: Kruskal–Wallis test to assess the effect of batch RNA extraction and the FFPE blocks’ age (years of blocks: 2003/2004/2005/2006/2007) on qRT-PCR expression of miRNAs, and genes selected for quality check purposes as they are highly expressed in tumors. Color codes indicate level of statistical significance. **Right**: Unsupervised hierarchical clustering of samples following gene expression analysis. Below the dendrogram, RNA quality parameters (i.e., year of sample inclusion and batch of RNA extraction) are depicted by colored bars.

Different aspects possibly affecting the quality of extracted RNA were considered—in particular, dependence from systematic effects, such as the extraction batch and FFPE block age. As shown in Figure 4, the extraction batch was the only variable affecting the quality of RNA extracted, in particular when quality was referred to gene expression analysis. This effect was particularly evident in MITO2 case material, while it was almost completely lost for the other three case materials (Figure 4, left panels). Following gene expression analysis, no evident clusterization of the samples was detected, considering the two pre-analytical systematic effects possibly influencing the quality of RNA (Figure 4, right panels).

## 4. Discussion

The search for diagnostic/prognostic/predictive biomarkers is still an urgent need in cancer research, and the best approach for successful biomarker discovery is to use bio-specimens derived from well-controlled clinical trials [3,8]. Many collections of ovarian cancer biospecimens have been used to characterize ovarian cancer from a prognostic point of view, with a general identification of four molecular subtypes, whose applicability in clinical practice is, however, still limited [9,10,11,12,13]. Efforts have also been made to identify predictive biomarkers using clinical trial-derived samples collected retrospectively [14,15,16,17]. However, a BRCA1/2 mutation or homologous recombination deficiency are the only biomarkers so far that are clinically applicable for ovarian cancer patients’ selection for targeted therapies [2,18,19]. 

The connection of personalized medicine development to clinical trials should be considered a high-priority [20]. The design of clinical trial with associated translational studies and the collection of high-quality tissue blocks with annotated clinical data are important steps forward for the easier translation of molecular discoveries to the bedside, as well as for the best selection of therapeutic interventions and the likelihood reduction that a trial produces negative results without mechanistic information [21]. However, the timing of a translational study’s design is extremely important. In fact, the design of translational studies after completion of the associated clinical trials necessary implies that the collection of biological material is not performed at study entry. As a consequence, the retrospective collection of samples might significantly reduce the possibility of including all the intention-to-treat population into the planned translational analysis [14,16]. Furthermore, in the case of randomized trials, a retrospective collection of FFPE blocks might also affect the balance between the treatment arms.

In collaboration with the Gynecological Cancer Inter Group (GCIG), we had already contributed by defining a roadmap to improve translational design for future gynecological cancer trials, in order to maximize patients’ benefit [8]. Here, we present the MITO model of coordinating translational activity in EOC clinical trials, which represents a unique experience in the area of Italian academic research. Considering that the MITO group is included in the GCIG group and the European Network for Gynecological Oncological Trial (ENGOT), this experience has been shared with the other trial groups discussing the problems related to sample collection, pathological revision, and quality controls for a mutual improvement of the procedures, and therefore to succeed with translational studies. 

This MITO experience surely had both strengths as well as limitations. Among its strengths, the first is that experts in different areas (clinical, translational, statistical, etc.) have been collaborating in an academic setting, sharing technologies and overcoming financial constraints. Moreover, the decision to centralize collection of biological samples, their pathological revision, and processing (TMA preparation and nucleic acids extraction) guarantied process standardization. Furthermore, quality assessment performed by qualified participating centers reduced the risks of possible biases, and allowed a quality double-check of the preparations. We also improved methodologies relative to the collection, processing, storage, and distribution of biospecimens for translational research purposes. A specific biological case report form (CRF) and dedicated SOP for the selection and shipping of FFPE blocks were included in the centralized database, starting with MITO7, since a certain number of samples were lost in MITO2 due to inconsistency of data with clinical information. This biological CRF is now incorporated into the studies. Among the limitations, we have to recognize that the retrospective collection of FFPE blocks for MITO2 and MITO7 projects resulted in a time-consuming process that greatly delayed the execution of proposed analyses, and in some cases negatively affected sample quality. This was particularly true for the MITO7 trial that was international. Therefore, one of our goals is to improve the timing of sample collection. In fact, for MITO16 trials, the collection of FFPE blocks was prospective and mandatory. This change in collection strategy significantly increased the numbers of FFPE centralized blocks (from 38% for MITO7 to 96% for MITO16A), and therefore, the number of patients included in translational analyses, although still not reaching 100% compliance. However, this limitation was also observed in other translational research experiences in EOC. Three papers and a presentation to the 2014 ASCO meeting presented data concerning translational studies associated with the two clinical studies that brought Bevacizumab into EOC first-line treatment: the Gynecologic Oncology Group (GOG) trial 218 [22] and the International Collaborative Ovarian Neoplasm (ICON) trial 7 [23]. From GOG 218, which enrolled 1248 patients, tumor microvessel density was evaluable on 980 FFPE samples (78.5% of the intended treatment population [14]), and plasma IL6 on 751 plasma samples collected at baseline (60% of enrolled patients) [16]. From the ICON7 trial, which enrolled 1528 patients, analyses were performed in the German and Edinburgh populations. The German group contributed 533 patients, of which gene expression analysis was evaluable for 359 patients (67% and 23.5% of the German cohort and of total population enrolled, respectively) [15]. The Edinburgh group contributed 387 cases, with gene expression analysis available for 265 (68.5% and 17% of the Edinburgh cohort, and 23.5% of total population enrolled) [17]. Overall, in our experience, even in prospective and mandatory sample collections, a low percentage of blocks were not made available, and a further 10% of drop-outs were a consequence of an accurate and centralized pathological revision that excluded blocks not complying with the inclusion criteria.

Further improvements have been introduced for the preparation of materials for the proposed analyses. For instance, TMA design changed according to the awareness of tumor heterogeneity, and in MITO16 studies, from each donor, three cores instead of two have been arrayed on recipient blocks. In addition, while IHC can be performed even in presence of a low percentage of tumor cells in the slide, to enable a good representation of the tumor heterogeneity, a higher percentage of tumor is generally required for miRNA or gene expression analyses. We decided to reach a threshold of 70% tumor cells and limit the possible necrotic area to20%, in the cases where RNA was extracted from whole sections. With the increase of the proposed translational analyses for MITO16 trials, RNA was extracted from tumor’s representative cores, instead from whole tumor sections; according to the imposed selection criteria, the number of FFPE blocks suitable for RNA extraction were lower than that for IHC. However, the extraction from cores ensured greater sternness in terms of percentage of cancer cells, and avoided the microtome cut or the need for blocks’ macrodissection with a faster and easier global process. A note of caution should be taken into consideration when molecular analyses such as RNA-seq are proposed from FFPE blocks. In fact, the formalin fixation process is known to create nucleic acids and protein cross-linkage, and RNA base methylation, thus preventing a reliable RNA-seq. However, due to efforts from companies producing reagents and protocols, and on the basis of recent technical advances [24,25], it is possible to envisage a future potential application of RNA-seq in the translational activities associated with prospective clinical trials. 

Despite the described limitations, in our experience the rigorous retrospective collection, pathological revision, processing, and association with well-controlled clinical data of FFPE blocks from the randomized MITO2 trial allowed the achievement of two important translational results. By profiling tumors for miRNA expression, we developed and validated an independent, prognostic, 35-miRNA-based predictor of risk of ovarian cancer relapse or progression (MiROvaR) identifying ovarian cancer patients at high risk of early relapse [26]. By IHC analysis, we identified a predictive value of DNA-PK and phosphorylated ACC expression [27]. The later result led to the design of a prospective, monocentric, pilot phase II validation study (use of new molecular markers for a personalized therapy in ovarian cancer, or MEMENTO) in one of the centers participating in the translational studies designed for MITO2.

We are aware that important margins of improvement to our strategies may be applied, and we summarized them in Table 4. 

Particular relevance should be given to a rigorous selection of the proposed translational analyses, to reduce the number of hypothesis generating explorative studies; the set-up of multi-parametric IHC analysis, to decrease the numbers of slides to be prepared; and a further optimization of timing of sample collection. In this context, an improvement has been introduced in the more recent and still ongoing MITO trials, in order to define the adequacy of tumor samples before patients’ accrual. In our experience, the procedure is feasible, and requires only a relative increase in the turnaround time for the patients to be treated. In particular, for an ongoing trial in ovarian cancer patients (MITO31; EUDRACT NUMBER 2018-000617-20), the mean turnaround time from the sample’s shipment to availability of the results at recruiting center was 5 days. 

Furthermore, the incidental presence of blocks collected during interval debulking surgery after the initiation of chemotherapy, together with synchronous primary and secondary tumor localizations, were considered added values for dedicated biological studies. In fact, the availability of biological samples longitudinally collected, or collected at different tumor sites, might shed light in the biology of ovarian cancer [28].

We are confident that our strategy, although with margins for improvement, may be a valuable example for the design of academic translational studies that, in the era of personalized medicine, are important and probably mandatory added values to clinical trials.

## Figures and Tables

**Table 1:** Selected MITO Clinical trials with translational end-points.

	MITO2	MITO7	MITO16A	MITO16B
**Type of trial**	First line Randomized phase III	First line Randomized phase III	Observational phase IV	Second line Randomized phase III
**Explorable biomarkers**	Prognostic and predictive	Prognostic and predictive	Prognostic only	Prognostic and predictive
**Samples collection and translational end-point**	Retrospective Secondary	Retrospective Secondary	Prospective Primary	Prospective Secondary
**N. centers and type of participation to TR analyses**	17/43 Voluntary	12/67Voluntary	47/47Mandatory	78/82Mandatory

TR: translational research.

**Table 2:** FFPE samples centralized from MITO clinical trials.

	N Pts. Enrolled in Clinical Trial	N Pts. with Approval for TR Analyses *	Patients with FFPE Blocks Centralized		
			Number	% Centralized/Enrolled	% Centralized/TR Approved *
MITO2	820	549	269	33%	49%
MITO7	810	457	176	22%	38%
MITO16A	400	400	385	96%	96%
MITO16B	406	406	366	90%	90%

Pts: patients. * Number of patients from centers with approval from Ethical Committees for biological studies (TR analyses).

**Table 3:** Synchronous primary tumor and secondary peritoneal samples.

Trial	Type of TR Analysis	Pts with Adequate Block	Pts with Primary Tumor Samples	Pts. with Synchronous Primary and Secondary Lesions	Pts. with Secondary Lesion Only
MITO2	IHC	239	230	52	8
	Molecular *	214	180	42	34
MITO7	IHC	158	127	49	31
	Molecular	136	108	41	28
MITO16A	IHC	358	252	58	106
	Molecular	290	223	41	67
MITO16B	IHC	313	180	31	133
	Molecular	256	165	26	91

TR: translational research; Pts: patients: IHC: immunohistochemestry. * miRNA and/or gene expression profiles.

**Table 4:** MITO translational activity: lessons learned and proposed future improvements.

Parameters	Limits	Solutions	Future Improvements
**Histological blocks’ retrival**	Blocks not available	Design of prospective clinical trial with associated translational studies	Further optimization of block collection timing
	Insufficient amount of primary tumor	Prospective evaluation of the block before patient enrollement	Rigorous selection of the proposed translational analyses to reduce the number of hypothesis generating explorative studies
	Assesment of spatial heterogeneity	Analysis on peritoneal secondary localizations	
**Histological blocks’ characteristics and processing**	Possible pathological diagnostic inconsintencies	Centralized pathological revision	Digital pathology for different pathologist evalution
	Unavailability of facilities and expertise	Centralized histological block processing (TMA preparation and nucleic acids extraction)	
	Definition of procedures	Preparation of specific biological CRF and dedicated SOPs for selection and shipping of FFPE blocks	
**IHC on whole sections**	Insufficient material	Reduction the number of hypothesis generating explorative studies	Multi-parametric IHC analysis to decrease the numbers of slides to be prepared
**TMA construction**	Awareness of tumor heterogeneity	TMA designed with 3 cores/samples instead of 2	
**RNA extraction and quality**	Need ≥70% of tumor cells and <20% of necrosis.	Tumor macrodissection	
	Tumor heterogeneity	Extraction from tumor-cores instead of tumor slices	
	RNA integrity	SOPs for samples fixation and storing	

## Supplementary Materials

The following are available online at https://www.mdpi.com/2073-4409/9/4/903/s1, Figure S1: Standard Operating Procedures for collection, shipping and processing of FFPE blocks developed and made available to MITO researchers through the coordinating center’s web-based platform. Figure S2: Flow diagram for retrospective collection of samples with translational purposes from MITO2 clinical trial. TMA: tissue macro-array; NACT: neo-adjuvant chemotherapy; IHC: immunohistochemistry. Figure S3: Flow diagram for retrospective collection of samples with translational purposes from MITO7 clinical trial. NACT: neo-adjuvant chemotherapy; IHC: immunohistochemistry. Figure S4: Flow diagram for prospective collection of samples with translational purposes from MITO16A/MaNGO-OV2 clinical trial. NACT: neo-adjuvant chemotherapy; IHC: immunohistochemistry. Figure S5: Flow diagram for prospective collection of samples with translational purposes from MITO16B/MaNGO-OV2B/ENGOT-OV17 clinical trial. NACT: neo-adjuvant chemotherapy; IHC: immunohistochemistry. Table S1: Criteria used for TMA construction.
