# Supplementary material for: Ovarian Cancer Translational Activity of the Multicenter Italian Trial in Ovarian Cancer (MITO) Group: Lessons Learned in 10 Years of Experience
Source: Cells. 2020 Apr 7;9(4):903. doi: 10.3390/cells9040903 (PMC7226822; doi:10.3390/cells9040903)
Supplement: Supplementary file 1 [file cells-09-00903-s001.pdf]

Ovarian cancer translational activity of Multicenter Italian Trial in Ovarian Cancer (MITO) group: lessons learned in 10 years of experience

Daniela Califano[1], et al.

SUPPLEMENTARY MATERIALS

RNA extraction and quality controls

From FFPE samples, RNA was extracted using Qiagen miRNeasy FFPE Kit with a partially modified protocol. Briefly, after proteinase K digestion, Qiacube (Qiagen), a robotic workstation for automated processing to standardized results and increase productivity, was used. When the starting material was the core of paraffin embedded tissues (MITO16 trials), after the deparaffinization a preliminary step of sample lysis was performed with Qiagen Tissue Lyser. RNA integrity was evaluated by real-time q-PCR amplification of housekeeping genes or miRNA considered constitutively over-expressed in tumors. In particular, different lengths of fragments of *ACTB* (actin beta) were amplified using TaqMan assays (ThermoFisher): hs010600665_g1 (63bp) and hs99999903_m1 (171bp). The difference $Ct_{63bp-171bp}$ was assessed; when $Ct_{63bp-171bp}$ is close to 0, the sample has low level of degradation, while the degradation increases if Ct63bp >> Ct171bp. The $Ct_{63bp-171bp}$ was implemented by Ct of *MRPL19* (mitochondrial ribosomal protein L19, hs00608519_m1) and *RPL13A* (ribosomal protein L13a, hs01926559_g1). A sample was considered as poor quality when: i) $Ct_{63bp-171bp}$ >8; ii) Ct *MRPL19* >30; Ct *RPL13A* >30. TaqMan gene primer sets were purchased from Life Technologies (LifeTechnologies, Carlsbad, CA, USA). Reverse transcription and PCR amplification for gene expression assays were performed with TaqMan Transcription and TaqMan Gene Expression Master Mix, respectively, following the manufacturer's instructions (LifeTechnologies, Carlsbad, CA, USA) and starting from 100 ng of total RNA. RT-qPCR data were normalized, using *POL2RA* as housekeeping gene. For miRNA, integrity check was performed by Ct of miR-21, miR-16, miR-103, miR-451. Starting from 20 ng total RNA, first strand cDNA was synthesized using miR-specific stem-loop primers and the High-Capacity cDNA Reverse Transcription Kit (Applied Biosystems); reactions were run in a GeneAmp PCR 9700 thermocycler (Applied Biosystems) at 16°C for 30 min, 42°C for 30 min, and 85°C for 5 min. The RT products, PCR master mix containing TaqMan 2× Universal PCR Master Mix (No Amperase UNG), and 10× TaqMan assay in 20 µL were amplified as follows: 95°C for 10 min, 40 cycles of 95°C for 15 sec, and 60°C for 60 sec. Gene and miRNA expression levels were quantified, using a sequence detection system (ABI Prism 7900HT; LifeTechnologies, Carlsbad, CA), and the

threshold cycle (Ct) for each sample was determined. ABI SDS 2.4 software (LifeTechnologies, Carlsbad, CA, USA) was used to recover the data.

SUPPLEMENTARY TABLES

Supplementary Table 1. Criteria used for TMA construction.

	MITO2	MITO7	MITO16A	MITO16B
**N° cores/Pts.**	2	2	3	3
**Centers randomization**	No*	Yes	Yes	Yes
**Cores randomization**	No	No	Yes	Yes

Pts: patients; *all the patients of each institution were in the same TMA

SUPPLEMENTARY FIGURES

TUMOUR TISSUE COLLECTION PROTOCOL MITO

Check the patient signature on the consent. **Obtain paper CRF Biological Sample Tumor tissue from www.usc-intnapoli.net**<br>Paper CRF serves as **source document** and tracks the specimen.	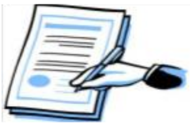
Obtain Formalin-fixed tumour tissue embedded in paraffin blocks from the primary tumour and/or a metastatic site if the primary tumour is unavailable, or if possible from both primary tumour and a metastatic site.	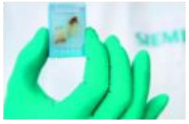
Obtain one haematoxylin/eosin stained slide.	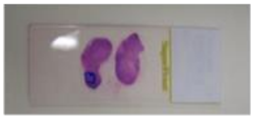
Obtain a copy of histology report and clearly write study name and study ID and blind out patient's name	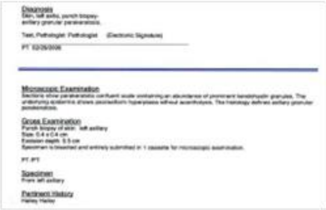
Complete the paper CRF and **store with study documents.**	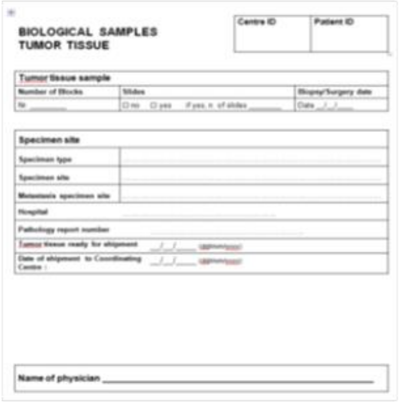<br>BIOLOGICAL SAMPLES<br>TUMOR TISSUE<br>Centre ID | Patient ID<br>Tumor tissue sample<br>Number of Blocks | Slides | Biopsy/Surgery date<br>Specimen site<br>Specimen type<br>Specimen site<br>Metastasis specimen site<br>Hospital<br>Pathology report number<br>Tumor tissue ready for shipment<br>Date of shipment to Coordinating Centre :<br>Name of physician
**Insert** all **data** in **eCRF**	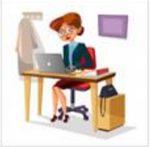
Prepare samples for shipment and include the **tumour block**, H/E stained slide, the histology report and complete specimen log. Clearly label the specimen package with Patient study ID.<br><br>Shipment will be scheduled by the coordinating center for every 10 patients randomized or every 2 months.	For information please contact:<br>Dr. :<br>Unit<br>Institute<br>Address:<br>Cap City<br>Tel<br>**Email:**

Supplementary Figure 1. Standard Operating Procedures for collection, shipping and processing of FFPE blocks developed and made available to MITO researchers through the coordinating center's web-based platform.

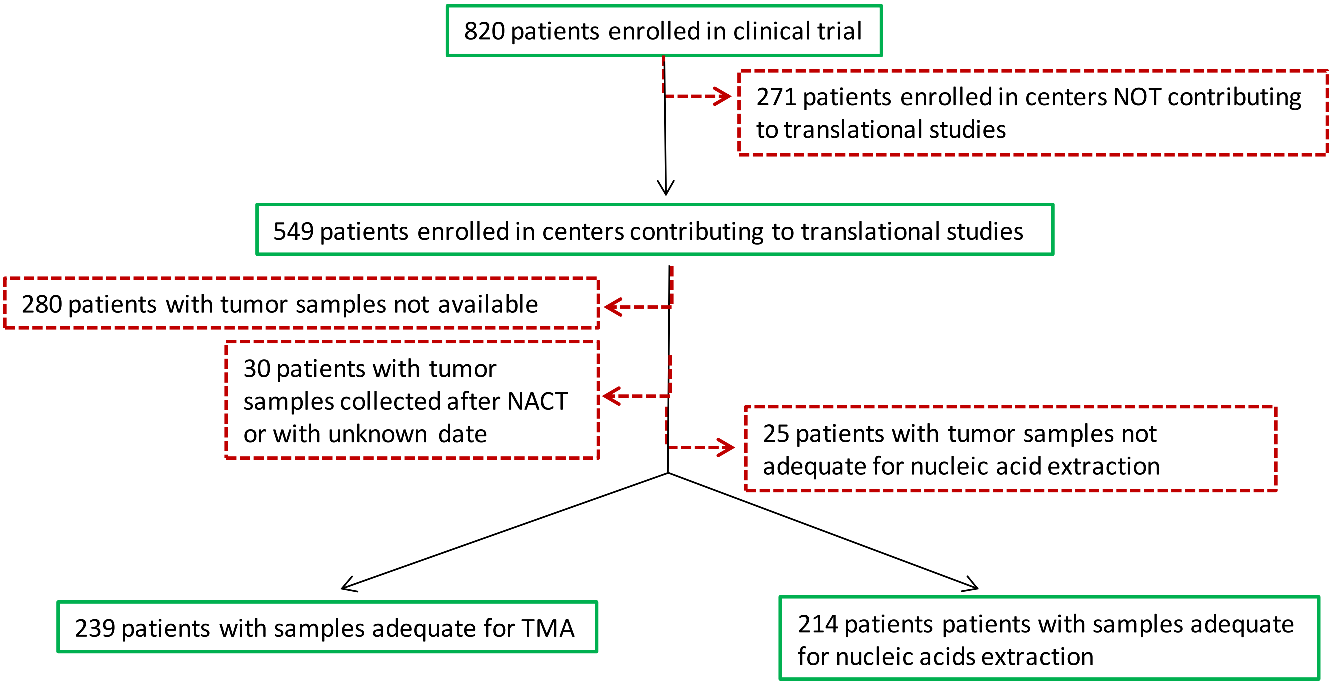

Supplementary Figure 2. Flow diagram for retrospective collection of samples with translational purposes from MITO2 clinical trial. TMA: tissue macro-array; NACT: neo-adjuvant chemotherapy; IHC: immunohistochemistry.

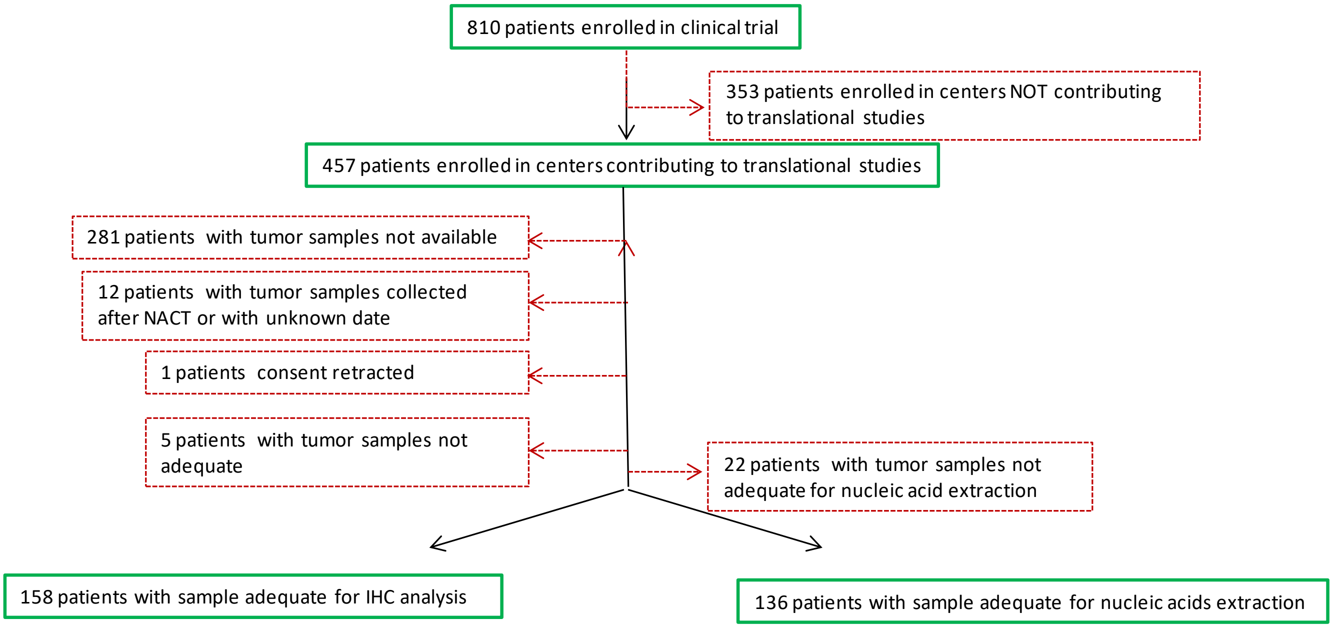

Supplementary Figure 3. Flow diagram for retrospective collection of samples with translational purposes from MITO7 clinical trial. NACT: neo-adjuvant chemotherapy; IHC: immunohistochemistry.

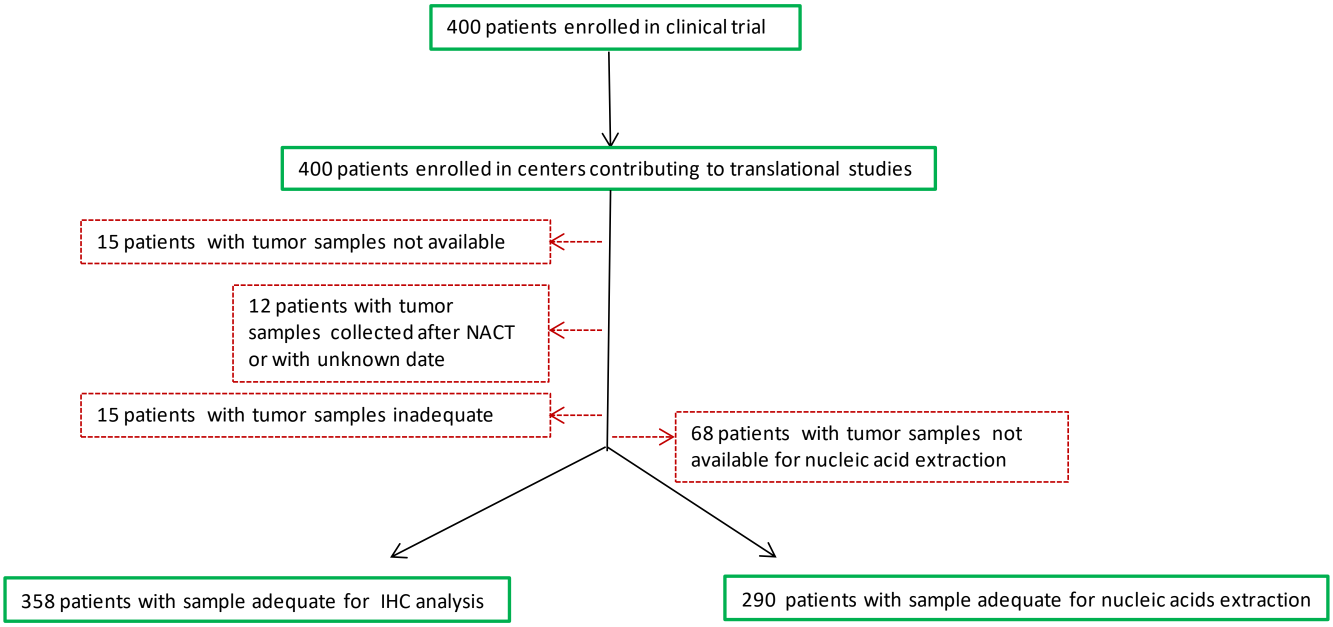

Supplementary Figure 4. Flow diagram for prospective collection of samples with translational purposes from MITO16A/MaNGO-OV2 clinical trial. NACT: neo-adjuvant chemotherapy; IHC: immunohistochemistry.

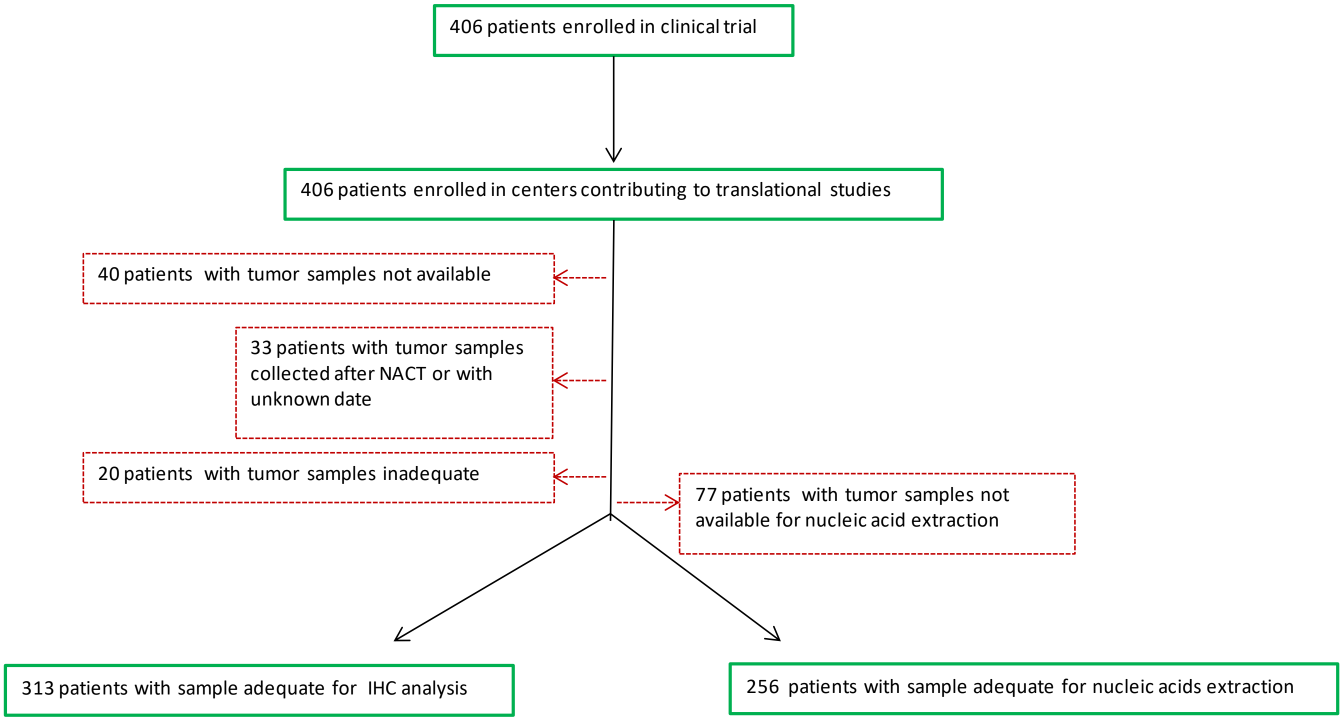

Supplementary Figure 5. Flow diagram for prospective collection of samples with translational purposes from MITO16B/MaNGO-OV2B/ENGOT-OV17 clinical trial. NACT: neo-adjuvant chemotherapy; IHC: immunohistochemistry.
